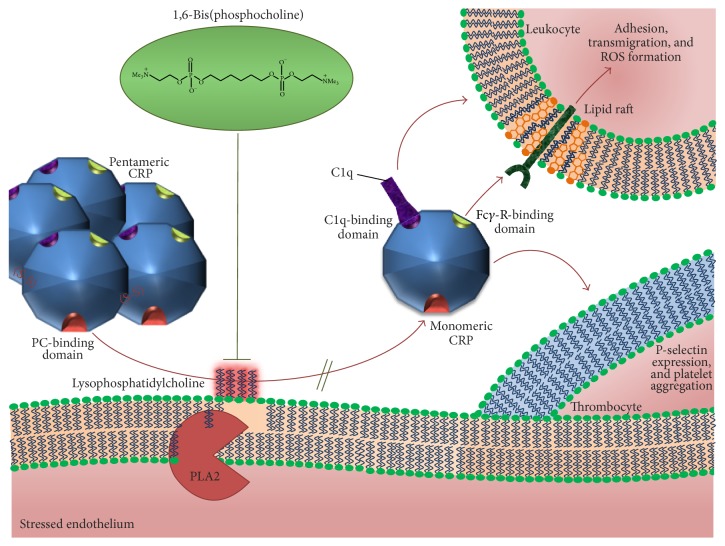# Targeting C-Reactive Protein in Inflammatory Disease by Preventing Conformational Changes

**DOI:** 10.1155/2015/372432

**Published:** 2015-05-18

**Authors:** J. R. Thiele, J. Zeller, H. Bannasch, G. B. Stark, K. Peter, S. U. Eisenhardt

**Affiliations:** ^1^Department of Plastic and Hand Surgery, University of Freiburg Medical Centre, Freiburg, Germany; ^2^Baker Heart and Diabetes Institute, Melbourne, VIC, Australia

## Abstract

C-reactive protein (CRP) is a pentraxin that has long been employed as a marker of inflammation in clinical practice. Recent findings brought up the idea of CRP to be not only a systemic marker but also a mediator of inflammation. New studies focused on structural changes of the plasma protein, revealing the existence of two distinct protein conformations associated with opposed inflammatory properties. Native, pentameric CRP (pCRP) is considered to be the circulating precursor form of monomeric CRP (mCRP) that has been identified to be strongly proinflammatory. Recently, a dissociation mechanism of pCRP has been identified on activated platelets and activated/apoptotic cells associated with the amplification of the proinflammatory potential. Correspondingly, CRP deposits found in inflamed tissues have been identified to exhibit the monomeric conformation by using conformation-specific antibodies. Here we review the current literature on the causal role of the dissociation mechanism of pCRP and the genesis of mCRP for the amplification of the proinflammatory potential in inflammatory reactions such as atherosclerosis and ischemia/reperfusion injury. The chance to prevent the formation of proinflammatory mediators in ubiquitous inflammatory cascades has pushed therapeutic strategies by targeting pCRP dissociation in inflammation. In this respect, the development of clinically applicable derivatives of the palindromic compound 1,6-bis(phosphocholine)-hexane (1,6-bis PC) should be a major focus of future CRP research.

## 1. Introduction

C-reactive protein (CRP) is a marker of inflammation that is extensively used in clinical practice. Recently, several prospective clinical studies have shown that modest elevations in baseline CRP levels predict future cardiovascular events [1–4]. This brought up the idea of CRP to be not only a systemic marker of inflammation but also a mediator in inflammatory foci.

CRP was discovered in Oswald Avery's laboratory at the Rockefeller Institute in New York City. William Tillett and Thomas Francis Jr. detected a protein in sera from patients with* Streptococcus pneumoniae* infection that interacted with pneumococcal cell wall residues. Increasing plasma concentrations of CRP as a result of tissue injury [5, 6] or inflammatory states [7–12] has been a long employed inflammatory parameter for clinical purposes. However, it took another forty years to identify the specific ligand for CRP, phosphocholine (PC) [13]. In the past, conflicting findings of the role of CRP in inflammation made it difficult to evaluate a potential involvement of CRP in the inflammatory cascade. Ideas of anti-CRP strategies became less attractive. However, recent studies suggested the existence of two conformations of the protein to explain the contradictory data. A dissociation mechanism of the pentameric protein (pCRP) to its monomeric subunits (mCRP) mediated by bioactive lipids [14] has been described and localized upon damaged and activated cells and platelets. This conformational change is accompanied with an alteration of the inflammatory profile of the protein [15]. The proinflammatory properties could now be attributed to the monomeric isoform and the dissociation process became the focus of anti-inflammatory therapeutic strategies.

Here, we review the recent literature of CRP as a mediator of inflammation and illustrate recent findings that reveal the crucial role of dissociation of pCRP and genesis of mCRP for the amplification of the proinflammatory potential in inflammatory reactions such as atherosclerosis and ischemia/reperfusion injury.

## 2. pCRP Is the Circulating Precursor Form of mCRP

### 2.1. Structure of Pentameric CRP

Pentameric C-reactive protein is part of the superfamily of pentraxins and as such consists of five identical, noncovalently associated globular protomers. 206 amino acids folded into two antiparallel *β*-sheets with flattened jelly-roll typology [16] forming one subunit of about 23 kDa molecular mass [17]. Each of the five subunits is linked by disulfide bonds [18] and is arranged symmetrically around a central pore composing a cyclic multimeric structure. Each protomer has been found to accommodate a hydrophobic pocket that represents the active site of binding PC. X-ray crystallography revealed the hydrophobic pocket on the so-called recognition face which consists of four amino acid residues. Especially Phe^66^ and Glu^81^ coordinate two calcium ions, mediating the binding of phosphocholine to CRP [19, 20].

The CRP-ligand phosphocholine is composed of one positively charged choline nitrogen head and a hydrophobic methyl group tail, whereat Phe^66^ of the binding site interacts with the tail and Glu^81^ provides ionic interaction with the head of PC. The effector face is located on the opposite side of the pentamer, in which the globular recognition domain of complement C1q binds and enables interaction with the classical complement pathway. To date, no mutation or deficiency of this phylogenetically highly conserved [21] plasma protein is known in human, suggesting a pivotal contribution to innate immune response.

### 2.2. Synthesis of Pentameric CRP

Pentameric CRP is predominantly expressed in hepatocytes and from there it is secreted into circulation [22]. An expression of pCRP has also been reported in neuronal cells [23], renal cortical tubular epithelial cells [24], arterial tissue, respiratory epithelium [25], adipocytes, and leukocytes [26–30]. However, it seems unlikely that extrahepatic synthesis affects plasma levels considerably. The proinflammatory cytokines interleukin 6 (IL-6) and, to a lesser extent, interleukin 1*β* (IL-1*β*) as well as tumor necrosis factor (TNF) induce CRP expression at the transcriptional level [31] through recruitment and activation of the transcriptional factors C/EBP*β* and C/EBP*δ*. Furthermore, STAT3 and Rel proteins (NF-*κ*) interact with gene regulation by binding to the proximal promoter region of the CRP gene, increasing the stability of C/EBP binding to the CRP gene, which results in maximum induction [32]. In contrast, both interferon-*α* (IFN-*α*) [33] and statins and nitric oxide in human hepatoma Hep3B cells suppress the induction of CRP expression by proinflammatory cytokines [34]. Thus, serum CRP levels poorly correlate with disease states that are associated with IFN-*α* signaling, such as viral infections or systemic lupus [35, 36]. Pentameric CRP is cleared from circulation and catabolized by hepatocytes* in vivo* and is not affected by inflammation and plasma concentration of pCRP, resulting in a half-life of 19–24 hours [37].

### 2.3. pCRP in Inflammation

During inflammation pCRP plasma levels can increase from undetectable levels in healthy individuals up to 1,000-fold and more within 24 to 72 hours [38]. Although baseline serum level elevations detected by high-sensitivity CRP assays are generally accepted to be a risk factor for developing cardiovascular disease [26, 39] and cancer [40]; a significant role of pCRP in the underlying pathological processes has been questioned [21, 40, 41]. This is in part because of the contradictory literature as both proinflammatory and anti-inflammatory effects of pCRP have been reported.

Pentameric CRP was suggested to upregulate the activation of DNA binding protein complex NF-*κ*B, a key mediator of atherosclerosis [42–44] and the expression of monocyte chemoattractant protein-1 (MCP-1) in human endothelial cells. NF-*κ*B regulates the activation of the gene encoding for the chemokine MCP-1 on the transcriptional level, which, in turn, promotes the migration of leukocytes into the subendothelial tissue and contributes to atherosclerosis. It was further postulated that pCRP induces the upregulation of cell adhesion molecules such as intercellular adhesion molecule-1 (ICAM-1), vascular cell adhesion molecule-1 (VCAM-1), and E-selectin via NF-*κ*B upregulation [45, 46].

However, proinflammatory properties that have been attributed to pCRP were found to be more likely due to contaminations of commercially available CRP preparations. C-reactive protein-induced* in vitro* vasorelaxation as well as proapoptotic effects in endothelial cells, for example, have been found to be an artefact caused by the presence of the commonly used preservative agent sodium azide [47, 48]. Corresponding to the contamination with the bacterial preservative sodium azide, endotoxin contamination with lipopolysaccharide (LPS) in recombinant CRP preparations provoked an acute phase response in mice, whereas purified pCRP did not [49]. The integrin Mac-1 (*α*_M_ *β*_2_; CD11b/CD18) can be used to rate monocyte activation. This receptor is expressed on monocytes and neutrophils and shows a function-specific conformation [50, 51]. However, pCRP failed to induce activation of Mac-1 on monocytes, monitored by binding of activation-specific anti-Mac-1 antibodies in flow cytometry, as well as fluorescence microscopy [52]. Khreiss et al. demonstrated that native pCRP has no effect on the overall expression of ICAM-1, VCAM-1, and E-Selectin in endothelial cells; however, treatment with mCRP significantly induced the expression of these adhesion molecules [53, 54]. Further investigations proved that the inflammatory response measured by neutrophil activation, adherence, and extravasation was attenuated when pCRP had been dialyzed prior to use [55, 56].

## 3. mCRP Represents the Proinflammatory Isoform of CRP

### 3.1. Genesis of Monomeric CRP

Native circulating pentameric CRP may dissociate under certain conditions that destabilize protein structure, such as exposure to heat, high concentrations of urea, acidic microenvironment [57, 58], prolonged storage in absence of calcium ions [59], or direct immobilization on polystyrene tissue culture flasks [57]. Though* in vitro* genesis of mCRP has been reported extensively, the* in vivo* existence has long been questioned. The molecular structure of pCRP has been described as extremely stable [60, 61] and protein denaturation has been seen as the only condition to generate the monomer [21]. However, Eisenhardt et al. demonstrated a dissociation process of pCRP to mCRP on activated platelets [52] which was then supported by reports of dissociation of pCRP after calcium ion-dependent binding to cell membranes and liposomes [14]. Only recently, the dissociation of circulating pCRP to mCRP after binding to activated endothelium in areas of inflammation could be observed* in vivo* in a rat model of acute inflammation [62]. This supports the findings of Habersberger et al. who postulated a pCRP dissociation mechanism on circulating microparticles in patients following myocardial infarction [63]. Strang et al. discovered a previously unrecognized potential of beta-amyloid plaques to dissociate pCRP to mCRP in the brains of patients with Alzheimer's disease and postulated an inflammatory role of mCRP in Alzheimer pathology [64].

However, these data have to be interpreted with care as alteration of the protein structure and even dissociation of pCRP may occur due to preparation of material for immunohistochemistry.

The dissociation process is mediated by exposure to lysophosphatidylcholine (LPC), a bioactive lipid, which is generated following phospholipase A2 (PLA2) expression on activated cell membranes [65]. These findings were confirmed by the* in vivo* application of the PLA2 inhibitor ONO-RS-082 that consequently prevented mCRP formation on activated cells [62]. The role of PLA2 enzymes as regulators of inflammation is supported by* in vivo* mouse models of ischemic brain injury [66]. Gonçalves et al. postulated a role for lipoprotein-associated, PLA2-generated LPC for human atherosclerotic plaque formation [67]. These findings combined with the previously described dissociation and localized deposition of mCRP in atherosclerotic plaques [52] link PLA2-mediated membrane changes and subsequent CRP dissociation in chronic inflammatory conditions such as atherosclerosis. The ability of CRP to aggravate the inflammatory response is thus dependent on PLA2-mediated membrane changes in localized inflammatory lesions as a prerequisite to dissociation and generation of mCRP. Electron microscopy revealed that the conformational change of pCRP after binding to membranes, including liposomes and cell membranes, first results in a partial structural change, producing molecules that express CRP subunit antigenicity, but with retained pentameric conformation. These molecules can then loose pentameric symmetry resulting in the well-recognized mCRP [14].

### 3.2. Solubility of Monomeric CRP

Monomeric CRP has been characterized by a decreased aqueous solubility due to a secondary protein structure shift from predominantly *β*-sheets to *α*-helices and expression of intersubunit contact residues, in particular the residues 197 to 202 [68]. In patients with high cardiovascular risk associated with increased pCRP levels, mCRP cannot be found in the peripheral circulation (unpublished observations, Eisenhardt et al.). Both nonreduced [69] and Cys-mutated mCRP were found to be rapidly cleared from the circulation after intravenous administration into mice [70].

Thus, mCRP rather represents the tissue-bound form of CRP as it has been detected in various tissues throughout the body [52, 71–78]. However, only recently, microparticles derived from stressed cells were found to both bind and transfer mCRP to activated endothelial cells, acting as a ferry in disseminating inflammation [63]. Through the process of membrane fusion, phagocytosis, or ligand engagement of mCRP loaded microparticles with cell surface [79], microparticles are capable of delivering a mCRP-based proinflammatory stimulus.

### 3.3. mCRP in Inflammation

Monomeric CRP is considered to be the inflammatory derivative of circulating pCRP. Some of the following findings are based on recombinant mCRP solutions expressed in* Escherichia coli*. Despite being purified to keep endotoxin levels below the detection limit, drawbacks of recombinant protein expression should be considered when interpreting the data. Interaction of mCRP with Fc*γ*-RIII (CD16) in human neutrophils and other receptors of the Fc*γ* family [80, 81] as well as lipid rafts microdomains on cell membranes [52, 82] are crucial for the mCRP-induced cellular signaling. As functional blockade of CD16 only partially inhibits the proinflammatory properties of mCRP on leukocytes and endothelial cells [52, 54, 68] alternative pathways have been proposed. Lipid rafts, cholesterol, and sphingolipids enriched microdomains have been identified for proinflammatory mCRP anchorage as human neutrophil activation through mCRP fails after disruption of lipid rafts by either methyl-*β* cyclodextrin or nystatin [82]. Cytokine release, generation of reactive oxygen species (ROS), and upregulation of the expression of adhesion molecules were absent in the treated group [82]. Using specific gene silencing, two major Fc*γ* receptors, Fc*γ*-RI (CD64), and Fc*γ*-RIII (CD16) could be identified as the major Fc*γ* receptors on human monocytes to mediate the proinflammatory potential independent of lipid raft signaling [62]. In neutrophils, mCRP attenuates DNA fragmentation through Fc*γ*-RIII (CD16) signaling, thus preventing apoptosis [68] similar to the antiapoptotic mediators granulocyte macrophage-colony stimulating factor, glucocorticoids, and LPS [83–85]. Khreiss et al. demonstrated that mCRP induces interleukin-8 (IL-8) secretion in human neutrophils via intracellular peroxynitrite (ONOO^−^) signaling and following activation of nuclear factor-*κ*B (NF-*κ*B) and activator protein-1 (AP-1) resulting as a major source of nitrosative stress [53]. The proinflammatory effect of mCRP is not restricted to leukocytes. The expression of the adhesion molecules ICAM-1, VCAM-1, and E-Selectin and the chemokines interleukin-8 (IL-8) and monocyte chemoattractant protein-1 (MCP-1) were found to be upregulated in human coronary artery endothelial cells (HCAEC) incubated with mCRP [54]. Conversely, prolonged culture was needed to detect endothelial cell activation after pCRP exposure. That could be attributed to dissociation of pCRP following activation of cell membranes.

Recent findings have shown that C1q complex colocalizes with mCRP in human frontal cortex sections of patients suffering from Alzheimer's disease (AD). The histological staining was performed with a conformation-specific antibody directed against mCRP [64]. Earlier histological studies have found that CRP and complement components (most often component C1q) can be found to be colocalized in myocardial tissue during acute myocardial infarction [86]. Consistent with these findings, animal studies have shown a complement-dependent increase in ischemic lesion size following infusion with CRP [87, 88].

However, mCRP interaction with the complement system differs depending on whether mCRP is in the ligand-free state or is immobilized on surface. Bound to surface either alone or colocalized with oxidized low-density lipoprotein (ox-LDL) or enzymatically modified low-density lipoprotein (E-LDL), mCRP enables the activation of the classical complement pathway. Via binding to C1q and following turnover of C3, immobilized mCRP has been suggested to recruit Factor H. Monomeric CRP-dependent complement activation thus bypasses the more inflammatory and destructive terminal sequence into membrane attack complex C5–9. In contrast, ligand-free mCRP exhibits an inhibitory activity towards complement, presumably by restricting the binding of C1q with other complement activators (e.g., antibody-coated ox-LDL) and thereby potentially protects unwanted complement activation in the fluid phase [89]. New* in vivo* studies have shown that the proinflammatory tissue-damaging effects of human CRP are dependent on complement system activity. This has been demonstrated in a model of myocardial infarction as well as in LPS-induced tissue injury by complement depletion through cobra venom factor [62].

Interestingly, it has been reported that both pCRP and C1q are found independently in circulation with no recognized interaction forming a regulatory safety mechanism. This can be explained by the fact that pCRP, in contrast to mCRP, cannot bind C1q and was found to be unable to activate the classical complement pathway in solution, when not bound to its ligand [90]. When complexed to its ligand phosphocholine, pCRP can bind C1q and can activate C1, which is further increased after disruption of the pentameric structure. The dissociation mechanism of pCRP upon activated cells represents an intermediate conversion step linking pCRP to complement activation and localizes activated complement components in areas of inflammation.

### 3.4. mCRP in Cardiovascular Disease

Local deposition of mCRP but not pCRP has been detected in infarcted areas of brain tissue in stroke patients [91] and in infarcted myocardial tissue of rats and humans [62]. In this regard, gentle preparation of tissue for detection of mCRP by immunohistochemistry as absence of extreme temperature or acidic pH values is a prerequisite for interpretation of these findings. Eisenhardt et al. showed that mCRP is the more potent reagent, both increasing monocyte activation and production of reactive oxygen species [50, 51]. Further analysis of THP1-monocytes indicated a proinflammatory alteration of the proteome induced by mCRP stimulation [92]. Monomeric CRP tested under physiological shear flow and in static models has been found to induce monocyte adhesion on various tissues [93]. Khreiss et al. reported opposing effects of C-reactive protein on shear-induced neutrophil-platelet adhesion. Whereas mCRP has been found to upregulate platelet P-selectin expression and neutrophil-platelet interaction, pCRP attenuated these key events of acute coronary syndrome [94]. This is in line with findings reporting that mCRP, unlike pCRP, has stimulatory effects on platelets [95] and facilitates thrombus growth via platelet stimulation [96].

In atheroma formation, monocyte arterial wall penetration and subsequent transformation into macrophages are considered to be crucial steps. Unlike pCRP, mCRP activates monocytes [52, 92] and colocalizes with macrophages in atherosclerotic plaques [52]. As mCRP has further shown to be highly prothrombotic [96], rupture of the fibrous cap separating the lesion from the arterial lumen may result in increased platelet aggregation [97].

As the majority of circulating microparticles (MP) have been found to be shed of activated thrombocytes [79, 98], a positive feedback mechanism in thrombus formation via CRP dissociation upon MPs derived from platelets can be assumed. Moreover, MP could activate mCRP in peripheral circulation, whereas potent platelets are stationary in regions of thrombus formation [63]. Suleiman et al. showed association in rise of circulating pCRP levels and size of acute myocardial infarction (MI) [99]. These results are even more interesting since Habersberger and colleagues detected higher mCRP on MPs in sera of patients presenting with a ST-elevation MI compared to a second group which had undergone percutaneous coronary intervention [63]. Monomeric CRP further accumulates complement component C1q [14, 90, 100], thereby contributing to ischemia/reperfusion injury in the myocardium.

Overall, these findings confirm the idea of CRP dissociation as a promising target to prevent proinflammatory amplification in acute (cardiac ischemia/reperfusion) and chronic (atherosclerosis) diseases.

## 4. mCRP Cannot Only Be Detected in Cardiovascular Disease

### 4.1. mCRP in Kidney Disease

Healthy renal tissue has been found to be negative for mCRP. However, Schwedler et al. detected mCRP deposition in diabetic patients with severe chronic kidney disease. The diabetic patients showed progressive tubular staining for mCRP associated with declining renal function and increasing severity of histologically detectable lesions. The authors proposed a local production of monomeric CRP, since mCRP staining was independent of proteinuria and tightly associated within the tubular cytoplasm [101].

### 4.2. mCRP in Chronic Neurodegenerative Disease

Strang et al. recently demonstrated that mCRP is associated with beta-amyloid (A-*β*) plaques in the cortical tissues from patients with Alzheimer's disease (AD). A-*β* plaques have been found to induce the dissociation of pCRP into individual monomers, whereas the nonaggregated peptide did not. Previous studies have shown the presence of CRP antigenicity in AD affected brain tissue [23, 102, 103]. However, Strang et al. were the first using conformation-specific antibodies to demonstrate that mCRP is colocalized with A-*β* in sections of the frontal cortex from patients with AD. The reported findings may link CRP to the inflammatory processes underlying the progression of AD [64].

### 4.3. mCRP in Rheumatic Disorders

Sjöwall et al. proposed that mCRP on the surface of apoptotic cell fragments could be the driving antigen for the production of anti-CRP antibodies in patients suffering from systemic lupus erythematosus (SLE) [104, 105]. Anti-CRP levels in sera from SLE patients have been found to correlate statistically significant with lupus disease activity. Intriguingly, these serum anti-CRP antibodies have been found not to be able to bind the circulating isoform pCRP, whereas immobilized mCRP is bound. The interaction of tissue-bound mCRP with anti-CRP antibodies in vascular tissue has been suggested to promote the production of atherosclerosis and could therefore link elevated anti-CRP serum levels with chronic vascular disease in SLE patients.

### 4.4. Targeting Monomeric C-Reactive Protein

The understanding of the dissociation mechanism as the underlying process in many inflammatory disorders may enable the development of novel therapeutic approaches by either inhibition of pCRP dissociation or inhibition of mCRP itself. As the dissociation of CRP is the more upstream process, the therapeutic blockade appears to be the more favorable approach. Blocking the dissociation of pCRP by cross-linking two pCRP molecules in a “double doughnut”-like complex, the palindromic compound 1,6-bis(phosphocholine)-hexane (1,6-bis PC) was found to abrogate proinflammatory properties. In a molar ratio of 5 1,6-bis PC : 2 pentameric CRP, it transfers the potentially inflammatory molecules to an inert complex, thereby inhibiting CRP interactions with complement and other proinflammatory ligands (e.g., phosphoethanolamine, modified LDL). 1,6-bis PC is a derivative of CRP-ligand phosphocholine (PC), and as such it is bound corresponding to phosphocholine in a calcium-dependent manner in the PC-binding pocket [106]. Recently, we demonstrated that the stabilization of CRP with 1,6-bis PC abolished mCRP formation and deposition* in vivo* [62] (Figure 1). Restrictively, 1,6-bis PC is not convenient for clinical purposes due to its pharmacokinetics and its low affinity to pCRP (*K*_*d*_ = 150 nM). After intravenous administration, 1,6-bis PC is rapidly cleared from circulation resulting in an approximated half-time of 90 min in mice [106]. Thus, a more potent drug with higher oral bioavailability, higher affinity to pCRP, and prolonged half-time needs to be designed to efficiently target the pCRP dissociation process as an innovative therapeutic strategy.

## 5. Conclusion

Currently available evidence suggests that mCRP has marked proinflammatory properties* in vitro* and* in vivo*. Activated membranes in acute and chronic inflammation thereby mediate the proinflammatory conformational change of the circulating pCRP and localize the proinflammatory monomer. This receptor-mediated process aggravates inflammation via leukocyte recruitment, endothelial activation, and recruitment of the complement cascade. 1,6-bis PC is able to inhibit the proinflammatory effects through stabilization of pCRP in a decameric form [63, 106], thereby inhibiting mCRP deposition. After successful prototype development, future studies will now have to focus on potential new compounds with improved oral bioavailability and a longer half-life to permit a potential anti-inflammatory clinical application.

## Figures and Tables

**Figure 1:**